# Changes in intra- and extracranial carotid plaque calcification: a 2-year follow-up study

**DOI:** 10.1038/s41598-023-34223-7

**Published:** 2023-05-24

**Authors:** T. Zadi, D. H. K. van Dam-Nolen, M. Aizaz, A. G. van der Kolk, P. J. Nederkoorn, J. Hendrikse, M. E. Kooi, A. van der Lugt, D. Bos

**Affiliations:** 1 Department of Radiology and Nuclear Medicine, Erasmus MC, University Medical Center Rotterdam, Doctor Molenwaterplein 40, 3015 GD Rotterdam, The Netherlands; 2 CARIM School for Cardiovascular Diseases, Maastricht University, Maastricht, The Netherlands; 3 Department of Radiology and Nuclear Medicine, Maastricht University Medical Center, Maastricht, The Netherlands; 4 Department of Radiology and Nuclear Medicine, University Medical Center Utrecht, Utrecht, The Netherlands; 5 Department of Radiology and Nuclear Medicine, Radboud University Nijmegen Medical Center, Nijmegen, The Netherlands; 6 Department of Neurology, Academic Medical Center, Amsterdam, The Netherlands; 7 Department of Epidemiology, Erasmus MC, University Medical Center Rotterdam, Rotterdam, the Netherlands

**Keywords:** Calcification, Carotid artery disease

## Abstract

Extra- and intracranial carotid plaque calcification might have plaque-stabilizing effects, yet information on changes in plaque calcification remains scarce. We evaluated changes in carotid plaque calcification over 2 years follow-up in patients with symptomatic carotid artery disease. This study is based on the PARISK-study, a multicenter cohort study, with TIA/minor stroke patients with ipsilateral mild-to-moderate carotid artery stenosis (< 70%). We included 79 patients (25% female, mean age 66 years) who underwent CTA imaging with 2 year interval. We assessed the volume of extra- and intracranial carotid artery calcification (ECAC and ICAC) and calculated the difference between baseline and follow-up ECAC and ICAC volume. We performed multivariable regression analyses to investigate the association between change of ECAC or ICAC with cardiovascular determinants. ECAC. We found increase (46.2%) and decrease (34%) in ECAC volume during 2 year follow-up, both significantly correlation with baseline ECAC volume (OR = 0.72, 95% CI 0.58–0.90 respectively OR = 2.24, 95% CI 1.60–3.13).We found significant correlation for change in ECAC volume with diabetes (β = 0.46, 95% CI 0.03–0.89) and baseline ECAC volume (β = 0.81, 95% CI 0.73–0.88). ICAC. We found increase (45.0%) and decrease (25.0%) in ICAC volume. The ICAC decrease was significantly correlated with baseline ICAC volume (OR = 2.17, 95% CI 1.48–3.16), age (OR = 2.00, 95% CI 1.19–3.38) and use of antihypertensive drugs (OR = 3.79, 95% CI 1.20–11.96]).The overall change of ICAC volume was also significantly correlated with diabetes (β = 0.92, 95% CI 1.59–7.02), use of oral hypoglycemic drugs (β = 0.86, 95% CI 0.12–1.59) and baseline ICAC volume (β = 0.71, 95% CI 0.55–0.87). We provide novel insights into the dynamics of carotid plaque calcification in symptomatic stroke patients.

## Introduction

Carotid atherosclerosis is an important risk factor for ischemic strokes^1^. The mechanism underlying this risk revolves around rupture of extracranial atherosclerotic plaques which results in thrombus formation and embolization of the thrombus into the distal intracranial arteries causing ischemia^2^. In determining which plaque is rupture-prone, or vulnerable, the composition of the plaque plays a pivotal role. In particular, calcification is of interest as larger amounts of calcification may exert potential plaque-stabilizing effects^3–5^.

Within the etiological framework of ischemic stroke, two sites within the carotid artery at which atherosclerotic disease often occurs are the carotid artery bifurcation (extracranial), and the carotid artery siphon (intracranial). Despite abundant data^6–8^ on the role of the presence and amount of calcification at these sites in the risk of ischemic stroke or stroke recurrence, data on the temporal dynamics of extra- and intracranial calcifications remain scarce. Yet, detailed insight into these dynamics, in terms of regression, progression or stabilization, and the determinants of these changes, may open novel pathways towards therapeutic or preventative strategies for stroke or stroke recurrence.

Given this background, we performed a 2-year follow-up study to investigate changes in and determinants of extra- and intracranial plaque calcification in patients with TIA or minor stroke.

## Materials and methods

### Study population

**Figure 1:**
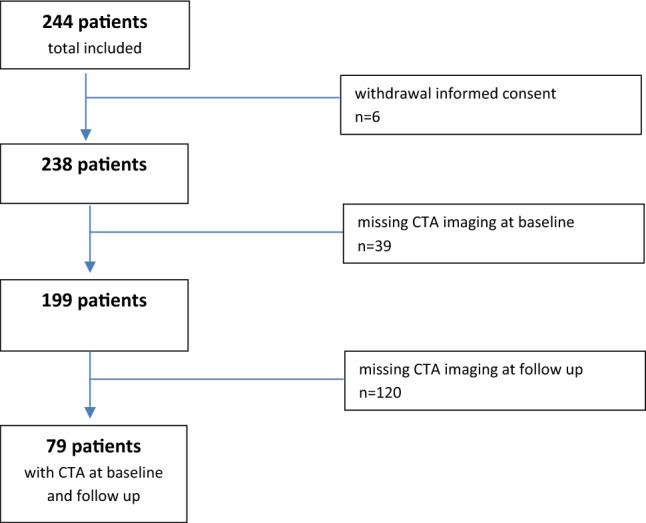
Flowchart of the study population.

This study is based on the PARISK study (Plaque At RISK study; clinical trials.gov NCT01208025), a prospective multicenter cohort study to identify patients with a high-risk for recurrent ischemic stroke with an ipsilateral mild-to-moderate carotid artery stenosis (< 70%). This study was approved by the institutional review board and all patients gave written informed consent. All included patients (n = 244) had a recent (< 3 months) TIA (including amaurosis fugax) or minor stroke in the carotid artery territory, prior to inclusion. Details of the study design are described previously^9^. For the current study, we included all participants who underwent computed tomography angiography (CTA) of the extracranial and intracranial carotid artery both at baseline and at 2 year follow-up (n = 79, Fig. 1).

### Assessment of carotid artery calcification

A 16, 64 or 128 slice multi-detector CT-scanner with standardized contrast-enhanced CTA protocols were used for visualization of the extra- and intracranial carotid arteries. Details of the imaging parameters are described previously^9,10^. The scan reached from the aortic arch to the intracranial vasculature (3 cm above the sella turcica). We measured extracranial carotid artery calcification (ECAC) bilaterally within 3 cm proximal (common carotid artery) and 3 cm distal to the carotid bifurcation (internal carotid artery) and intracranial carotid artery calcification (ICAC) bilaterally from the horizontal petrous segment until the circle of Willis.

For quantification of the calcification volumes we used a custom-made plug-in for the freely available Image J software (National Institutes of Health, Bethesda, Maryland). Calcification volumes were calculated as the number of pixels above a threshold of 600 HU multiplied by the slice increment and were expressed in mm^3^. This threshold was used to differentiate calcification from contrast material in the carotid artery. Further description of the measurements is provided elsewhere^11^.

### Assessment of demographic and cardiovascular risk factors

Clinical data such as age, sex, cardiovascular risk factors and medication use were collected at baseline. Details of the definition of cardiovascular risk factors (diabetes mellitus, hypertension, hypercholesterolemia, BMI, and current smoker) are described previously^9,10^. We defined history of cardiovascular disease (CVD) as the presence of a history of ischemic stroke, TIA, ischemic heart disease, peripheral artery disease, or other vascular diseases.

### Statistical analysis

To describe changes in ECAC and ICAC, and to investigate determinants of these changes, we used the following strategy.

First, we calculated absolute and relative differences in calcification volume for ECAC and ICAC. Absolute differences were calculated by subtracting the baseline calcification volumes from those at follow-up. Relative differences were calculated as (follow-up volume − baseline volume)/baseline volume × 100%. Second, we created three groups based on the changes in calcification, namely “calcification increase”, “stable calcification”, and “calcification decrease”. Importantly, this categorization was based on the relative change with a threshold of 10% to account for measurement error at both time points, to deal with interscan and interobserver variability. Third, we used a two-step approach to investigate associations of cardiovascular risk factors with change in calcification. We investigated the association with (1) increase and decrease in calcification volume using multivariable logistic regression models, and (2) follow-up calcification volume using multivariable linear regression models. Since the change in calcification volumes showed a non-normal distribution, we naturally log-transformed them after adding 1.0 mm^3^ to the volumes (to deal with calcification volumes of 0). We performed adjusted analysis for age, sex, ECAC or ICAC volume at baseline and time between CTA scans. We used a generalized estimation equation (GEE) approach to deal with the in-patient correlation, knowing that one patient has two arteries with the same underlying cardiovascular risk factors (patient cluster). Statistical analyses were performed using R statistical software (version 3.4.2).

### Ethics approval

This study was performed in line with the principles of the Declaration of Helsinki. Approval was granted by the Ethics Committee of Academic Medical Center Maastricht (NL29116.068.09/MEC 09-2-082).

### Consent to participate

Informed consent was obtained from all individual participants included in the study.

## Results

Seventy-nine patients were included. Clinical and imaging characteristics of the study population are described in Tables 1 and 2. Mean age was 66 ± 8 years and 75% of the patients were males. The two most important cardiovascular risk factors at baseline were hypertension and hypercholesterolemia with a prevalence of 76.8% and 79.5%.

**Table 1:** Clinical characteristics per patient.

Variable	Total (*n* = 79)	Males (*n* = 59)	Females (*n* = 20)
Clinical characteristics			
Age, years	66 ± 8	66 ± 8	64 ± 9
Diabetes mellitus, *n* (%)	14 (19.2%)	12 (21.4%)	2 (11.8%)
Hypertension, *n* (%)	53 (76.8%)	42 (77.8%)	11 (73.3%)
Hypercholesterolemia, *n* (%)	62 (79.5%)	47 (79.7%)	15 (79.0%)
BMI, kg/m^2^	26 ± 4	26 ± 4	26 ± 4
Current smoker, *n* (%)	22 (27.9%)	14 (23.7%)	8 (40.0%)
History of CVD, *n* (%)	41 (51.9%)	35 (59.3%)	6 (30.0%)
Medication use at discharge			
Use of oral hypoglycemic drugs at discharge, *n* (%)	12 (15.2%)	11 (18.6%)	1 (5.0%)
Use of antihypertensive drugs at discharge, *n* (%)	48 (60.8%)	39 (66.1%)	9 (45.0%)
Use of lipid-lowering drugs at discharge, *n* (%)	70 (88.6%)	54 (91.5%)	16 (80.0%)

Continuous variables are denoted by mean ± standard deviation. *BMI* body mass index, *CVD* cardiovascular disease. Data are missing for diabetes mellitus (*n* = 6), hypertension (*n* = 10), hypercholesterolemia (*n* = 1), BMI (*n* = 1).

**Table 2:** Imaging characteristics per artery.

Extracranial carotid artery	Total (*n* = 135/156)	Male (*n* = 101/116)	Female (*n* = 34/40)
Calcification volumes at baseline			
Calcification volume left, mm^3^	27.7 [11.3; 67.0]	27.4 [9.9; 65.9]	35.6 [16.5; 67.0]
Calcification volume right, mm^3^	32.8 [9.9; 89.0]	31.9 [7.0; 92.4]	39.7 [21.0; 73.4]
Calcification volume total, mm^3^	55.8 [24.7; 152.4]	53.0 [16.4; 171.3]	60.9 [41.0; 137.7]
Calcification volumes at 2 year follow-up			
Calcification volume left, mm^3^	39.1 [14.0; 86.3]	32.7 [13.5; 63.5]	40.8 [16.8; 92.2]
Calcification volume right, mm^3^	42.6 [10.1; 73.2]	42.9 [17.4; 91.9]	40.3 [6.0; 62.6]
Calcification volume total, mm^3^	72.1 [34.1; 161.0]	60.4 [34.0; 179.3]	94.1 [44.9; 145.5]

Intracranial carotid artery	Total (*n* = 99/140)	Male (*n* = 73/100)	Female (*n* = 26/40)
Calcification volumes at baseline			
Calcification volume left, mm^3^	17.8 [5.2; 52.8]	17.9 [5.1; 58.9]	15.3 [5.6; 27.6]
Calcification volume right, mm^3^	17.3 [6.7; 77.3]	18.2 [6.4; 77.3]	15.6 [11.1; 48.3]
Calcification volume total, mm^3^	36.1 [8.0; 120.9]	40.3 [7.2; 123.3]	34.6 [17.3; 47.3]
Calcification volumes at 2 year follow-up			
Calcification volume left, mm^3^	21.5 [6.1; 42.9]	24.2 [7.3; 52.3]	12.7 [3.9; 27.8]
Calcification volume right, mm^3^	20.7 [7.1; 46.0]	25.6 [11.7; 46.2]	10.3 [4.0; 27.7]
Calcification volume total, mm^3^	37.2 [12.5; 85.7]	40.4 [16.4; 94.4]	19.6 [10.1; 51.1]

Variables are denoted by median [interquartile range]. Extra- and intracranial calcification volume total represent the sum of the calcification volumes at the left and right side. n = x/x: total extra- or intracranial carotid arteries with presence of plaque calcification/total included extra- or intracranial arteries.

### ECAC volume

ECAC was bilaterally evaluated in 78 of the 79 patients. One patient was excluded due to an occlusion distal to the carotid bifurcation. The median total calcification volume at baseline was 55.8 mm^3^ [interquartile range (IQR) = 24.7; 152.4] and increased to 72.1 mm^3^ [IQR = 34.1; 161.0] at 2 year follow-up (Table 2).

**Figure 2:**
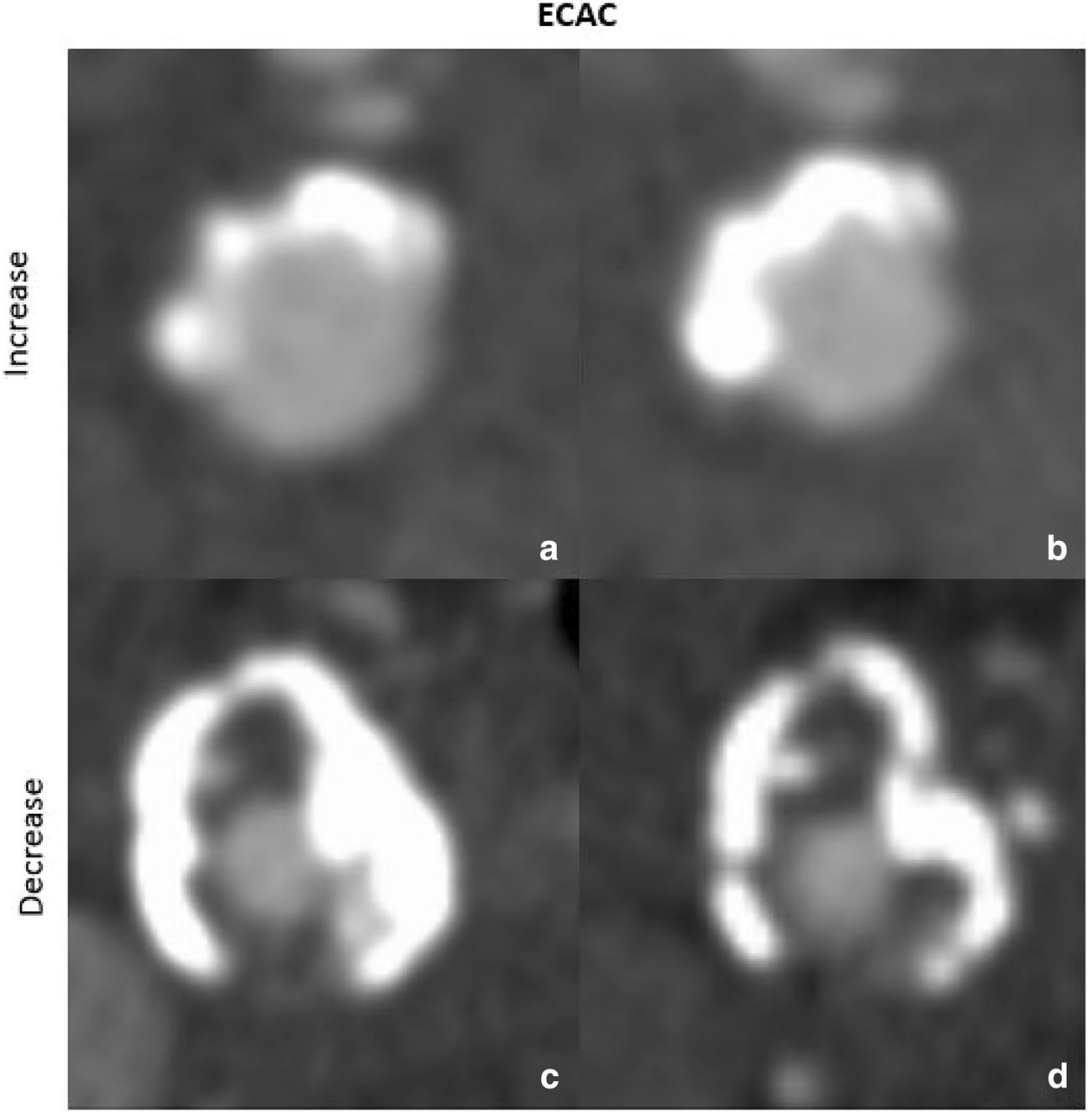
Increase and decrease of extracranial calcification volumes at baseline ((**a**) 201.2 mm^3^ and (**c**) 316.6 mm^3^) and follow-up ((**b**) 1235.6 mm^3^ and (**d**) 162.3 mm^3^).

ECAC volume showed an increase (Fig. 2a,b) in 72 of 156 arteries (46.2%), with an absolute volume increase of 23.0 mm^3^ [IQR = 3.9; 40.1] and a decrease (Fig. 2c,d) in 53 of 156 arteries (34.0%), with an absolute volume decrease of − 36.4 mm^3^ [IQR = − 78.4; − 10.0] (Suppl. Table 1).

Table 3 presents the correlation between cardiovascular risk factors and ECAC increase or decrease. Both ECAC increase and ECAC decrease were associated with ECAC volume at baseline after adjustments for age, sex, scan interval, and patient cluster (adjusted OR per Ln increase for ECAC increase = 0.72, 95% CI 0.58–0.90; adjusted OR per Ln increase for ECAC decrease = 2.24, 95% CI 1.60–3.13).

**Table 3:** Multivariable logistic regression analyses for change in ECAC.

Variable	Increased ECAC OR [95% CI]	*P* value	Decreased ECAC OR [95% CI]	*P* value
Clinical characteristics				
Female sex	0.98 [0.42; 2.32]	0.96	0.95 [0.35; 2.56]	0.92
Age, per 10 years	1.18 [0.77; 1.82]	0.44	1.22 [0.78; 1.89]	0.38
Diabetes mellitus	1.00 [0.38; 2.68]	0.99	1.29 [0.45; 3.73]	0.63
Hypertension	1.06 [0.43; 2.61]	0.91	0.96 [0.36; 2.58]	0.94
Hypercholesterolemia	0.99 [0.38; 2.55]	0.98	1.70 [0.58; 4.98]	0.34
BMI	1.06 [0.97; 1.15]	0.18	0.97 [0.89; 1.07]	0.54
Current smoker	0.92 [0.39; 2.15]	0.84	0.70 [0.28; 1.75]	0.44
History of CVD	1.27 [0.57; 2.82]	0.56	0.64 [0.28; 1.48]	0.30
Medication use at discharge				
Oral hypoglycemic drugs	0.78 [0.26; 2.33]	0.65	1.47 [0.47; 4.62]	0.51
Antihypertensive drugs	0.68 [0.31; 1.48]	0.33	1.15 [0.49; 2.72]	0.75
Lipid-lowering drugs	0.98 [0.28; 3.46]	0.98	1.76 [0.42; 7.40]	0.44
Calcification volumes at baseline				
ECAC volume	0.72 [0.58; 0.90]	0.004	2.24 [1.60; 3.13]	< 0.001

Values represent odds ratios [95% confidence intervals]. Dependent variable is increase or decrease of extracranial calcification volume. Independent variables are clinical and imaging characteristics. *ECAC* extracranial carotid artery calcification, *BMI* body mass index, *CVD* cardiovascular disease. Analyses are adjusted for age, sex, scan interval and patient cluster. Baseline calcification volumes are Ln transformed.

ECAC volume at 2 year follow-up was correlated with diabetes mellitus (β = 0.46, 95% CI 0.03–0.89) after adjustment for age, sex, ECAC volume at baseline, scan interval, and patient cluster, and was also associated with ECAC volume at baseline (β = 0.81, 95% CI 0.73–0.88]), after adjustment for age, sex, scan interval, and patient cluster (Table 4).

**Table 4:** Multivariable linear regression analyses for follow-up calcification volume.

Variable	ECAC volume at 2 year follow-up (*n* = 156)	*P* value	ICAC volume at 2 year follow-up (*n* = 140)	*P* value
Clinical characteristics				
Female sex	− 0.20 [− 0.67; 0.27]	0.41	− 0.46 [− 1.01; 0.09]	0.10
Age, per 10 years	0.22 [− 0.04; 0.48]	0.10	0.02 [− 0.26; 0.30]	0.90
Diabetes mellitus	0.46 [0.03; 0.89]	0.04	0.92 [0.24; 1.59]	0.01
Hypertension	0.01 [− 0.48; 0.50]	0.98	0.00 [− 0.41; 0.40]	0.99
Hypercholesterolemia	0.05 [− 0.57; 0.66]	0.88	− 0.12 [− 0.75; 0.50]	0.70
BMI, kg/m^2^	0.05 [0.00; 0.09]	0.05	0.02 [− 0.07; 0.11]	0.70
Current smoker	− 0.16 [− 0.57; 0.26]	0.47	− 0.33 [− 0.95; 0.29]	0.30
History of CVD	0.17 [− 0.18; 0.52]	0.33	− 0.11 [− 0.56; 0.35]	0.65
Medication use at discharge				
Oral hypoglycemic drugs	0.43 [− 0.05; 0.92]	0.08	0.86 [0.12; 1.59]	0.02
Antihypertensive drugs	0.02 [− 0.37; 0.40]	0.93	0.06 [− 0.35; 0.47]	0.77
Lipid-lowering drugs	− 0.27 [− 0.90; 0.36]	0.41	0.15 [− 0.37; 0.67]	0.57
Calcification volumes at baseline				
ECAC volume total at baseline, mm^3^	0.81 [0.73; 0.88]	< 0.001		
ICAC volume total at baseline, mm^3^			0.71 [0.55; 0.87]	< 0.001

Values represent beta’s [95% confidence intervals]. Dependent variable is the extra- or intracranial calcification volume at 2 year follow-up. Independent variables are clinical and imaging characteristics. Analyses are adjusted for age, sex, EC or IC calcification volume at baseline, scan interval and patient cluster. Baseline and follow-up calcification volumes are Ln transformed. *ECAC* extracranial carotid artery calcification, *ICAC* intracranial carotid artery calcification, *BMI* body mass index, *CVD* cardiovascular disease.

### ICAC volume

ICAC was bilaterally evaluated in 70 of the 79 patients. Nine patients were excluded due to incomplete imaging of the intracranial carotid artery. The median total calcification volume at baseline was 36.1 mm^3^ [IQR = 8.0; 120.9] and increased to 37.2 mm^3^ [12.5; 85.7] at 2 year follow-up (Table 2).

**Figure 3:**
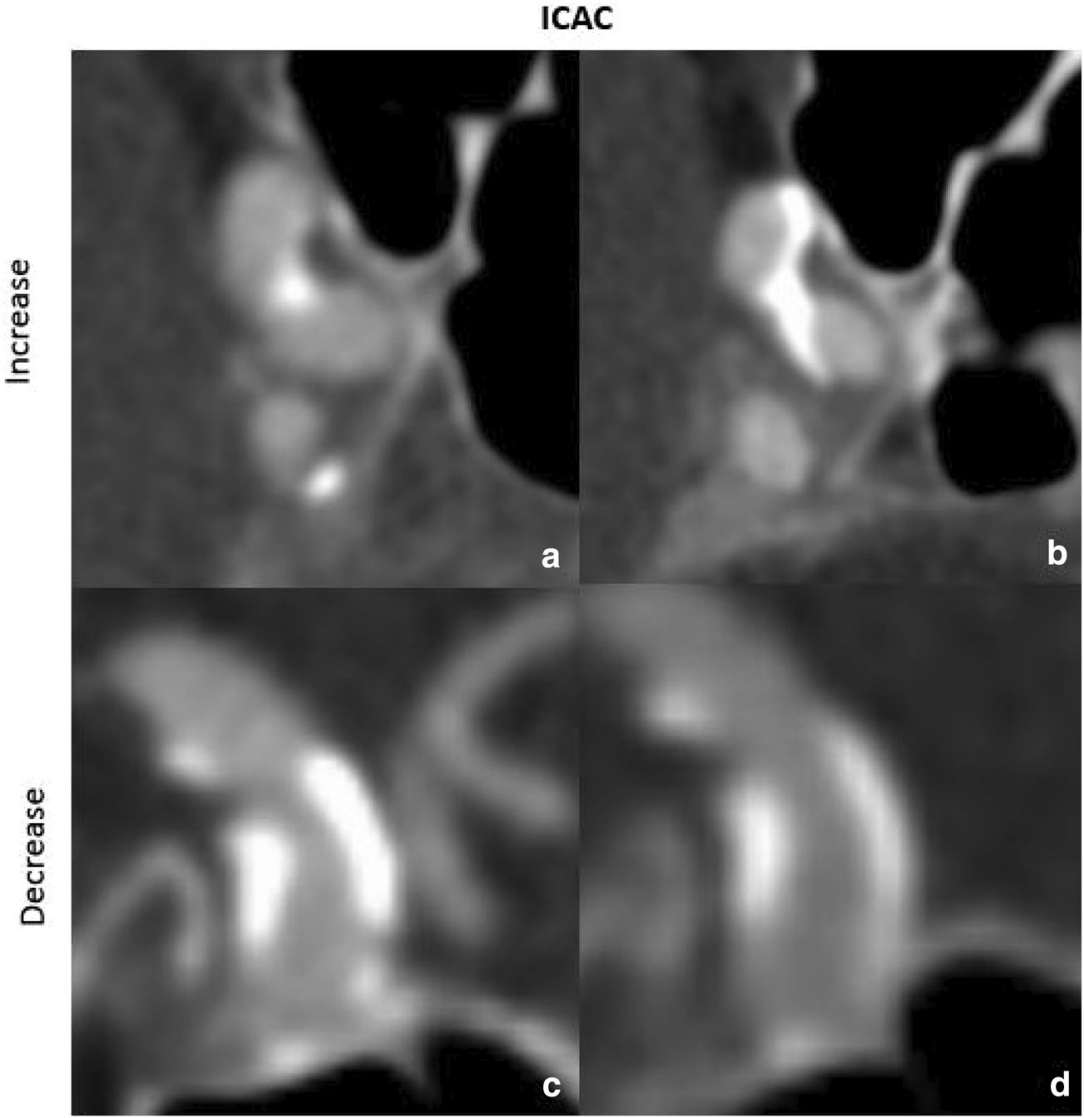
Increase and decrease of intracranial calcification volumes at baseline ((**a**) 49.5 mm^3^ and (**c**) 337.7 mm^3^) and follow-up ((**b**) 254.9 mm^3^ and (**d**) 11.7 mm^3^).

ICAC volume showed an increase (Fig. 3a,b) in 63 of 140 arteries (45.0%), with an absolute volume increase of 12.9 mm^3^ [IQR = 7.2; 27.5] and decrease (Fig. 3c,d) in 35 of 140 arteries (25.0%), with an absolute volume decrease of − 32.8 mm^3^ [IQR = − 88.7; − 11.5] (Suppl. Table 1).

None of the cardiovascular risk factors were statistically significant associated with increased ICAC (Table 5). Age (adjusted OR per 10 year increase = 2.00, 95% CI 1.19–3.38), use of antihypertensive drugs (adjusted OR = 3.79, 95% CI 1.20–11.96) and ICAC volume at baseline (adjusted OR per Ln increase = 2.17, 95% CI 1.48–3.16) were associated with decreased ICAC.

**Table 5:** Multivariable logistic regression analyses for change in ICAC.

Variable	Increased ICAC OR [95% CI]	*P* value	Decreased ICAC OR [95% CI]	*P* value
Clinical characteristics				
Female sex	1.12 [0.44; 2.89]	0.81	1.01 [0.32; 3.23]	0.98
Age, per 10 years	1.05 [0.62; 1.77]	0.87	2.00 [1.19; 3.38]	0.01
Diabetes mellitus	1.98 [0.61; 6.40]	0.25	2.00 [0.57; 6.96]	0.28
Hypertension	0.71 [0.23; 2.20]	0.55	3.54 [0.87; 14.48]	0.08
Hypercholesterolemia	0.91 [0.29; 2.89]	0.87	0.90 [0.19; 4.18]	0.89
BMI	0.97 [0.84; 1.12]	0.63	1.05 [0.90; 1.22]	0.55
Current smoker	0.59 [0.20; 1.74]	0.34	1.47 [0.45; 4.83]	0.53
History of CVD	0.64 [0.25; 1.64]	0.35	1.10 [0.37; 3.29]	0.86
Medication use at discharge				
Oral hypoglycemic drugs	1.51 [0.44; 5.26]	0.51	2.27 [0.63; 8.11]	0.21
Antihypertensive drugs	0.86 [0.34; 2.16]	0.75	3.79 [1.20; 11.96]	0.02
Lipid-lowering drugs	1.72 [0.41; 7.24]	0.46	2.43 [0.23; 26.23]	0.46
Calcification volumes at baseline				
ICAC volume	0.91 [0.72; 1.14]	0.41	2.17 [1.48; 3.16]	< 0.001

Values represent odds ratios [95% confidence intervals]. Dependent variable is increase or decrease of intracranial calcification volume. Independent variables are clinical and imaging characteristics. *ICAC* intracranial carotid artery calcification, *BMI* body mass index, *CVD* cardiovascular disease. Analyses are adjusted for age, sex, scan interval and patient cluster. Baseline calcification volumes are Ln transformed.

ICAC volume at 2 year follow-up was correlated with diabetes mellitus (β = 0.92, 95% CI 0.24–1.59) and with use of oral hypoglycemic drugs (β = 0.86, 95% CI 0.12–1.59) after adjustment for age, sex, ICAC volume at baseline, scan interval and patient cluster, and associated with ICAC volume at baseline (β = 0.71, 95% CI 0.55–0.87) after adjustment for age, sex, scan interval and patient cluster (Table 4).

**Figure 4:**
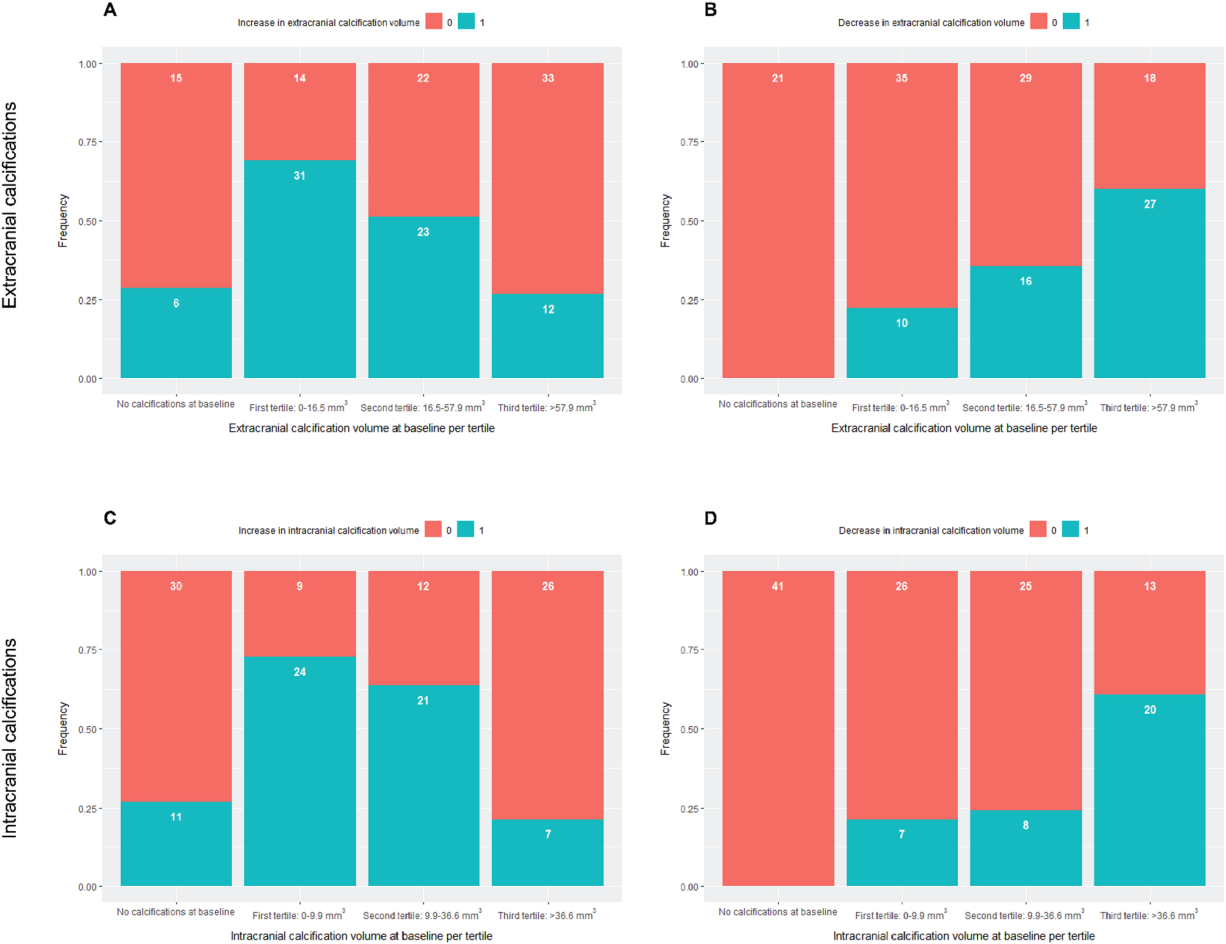
Change in calcification volume per tertile.

Figure 4 shows the proportion of increase and decrease in ECAC and ICAC volume per tertile of calcification volume at baseline. The diagrams illustrate a higher prevalence of increase in calcification volume (for both ECAC and ICAC) for a lower calcification volume at baseline. In addition, a higher prevalence of decrease was found in arteries with a higher calcification volume at baseline.

## Discussion

In this study, we evaluated the natural course of atherosclerotic plaque calcification in the intra- and extracranial part of the carotid artery over 2 year follow-up. Overall, a progression was seen in a narrow majority, which was associated with a lower calcification volume at baseline. Remarkably, we also found regression in calcification volume, which was associated with a higher calcification volume at baseline.

This study showed in a narrow majority of the study population, progression of plaque calcification in the internal carotid artery, both extra- (46% patients) and intracranial (45% patients). Our results show that plaque calcification can develop in different ways: increase, decrease or stabilization (with no or minimum change over time).

Surprisingly, we also found decrease of plaque calcification in both extra- and intracranial carotid arteries. ECAC decrease was statistically significant associated with baseline ECAC volume, and ICAC decrease with age, antihypertensive drugs and baseline ICAC volume. The fact that calcification can decrease over time is to our knowledge a novel finding. We saw more decrease in patients with specific (cardiovascular) risk factors. The highest decrease was seen in two patients with hematologic cancer treated with chemotherapy. We hypothesize that the use of anti-inflammatory medication could play a role in this process. Based on previous literature, we already know that anti-inflammatory medication might delay atherosclerosis progression^9^, yet it may also cause decrease of calcification. We recommend to investigate this finding of decreasing calcification further and more deeply in future research, first to confirm our results in other populations and secondly to identify potential pathophysiological mechanisms. It also is important to investigate whether an increase or decrease would be beneficial or harmful with regard to risk of (recurrent) stroke.

We have used total calcification volume. However, previous studies identified different subtypes of plaque calcification, with each a different risk of (recurrent) stroke. Subtypes based on size are micro-, spotty and large calcification. Microcalcification (between 0.5–50 µm) is correlated with inflammation within the active phase of atherosclerosis^12,13^. This leads to a higher macrophage volume, which shifts the stress concentration within the plaque towards the vessel lumen and can cause plaque rupture^14^. However, microcalcification is undetectable with CTA, due to a current spatial resolution below the threshold needed for identification. A higher inflammatory burden is also seen in plaques with spotty calcification (calcium deposits of < 4 mm, within an arc of less than 90°)^15^. Based on this theory, if a higher volume of calcification is not a large calcification (arc of > 90°), it would lead to more inflammation contrary to a stable plaque^14,16,17^. Subtypes of calcification might give insight in the development (including decrease) of calcification volume. Therefore, it is highly recommended for further research.

Furthermore, we found a statistically significant correlation with diabetes mellitus and calcification volume after 2 year follow-up. This correlation was seen for both ECAC and ICAC (β = 0.46, 95% CI 0.03–0.89 and β = 0.92, 95% CI 1.59–7.02). We hypothesize that this correlation is caused by a higher inflammatory burden, due to endothelial damage of the small vessels as a result of diabetes mellitus. Related to this active phase, we think subsequently an increase of micro- or spotty calcification would be more likely to occur, resulting in increasing calcification volume^18^. Van Gils et al. who also investigated changes in calcification during follow-up found more statistically significant associations with cardiovascular risk factors. An explanation why these associations were not statistically significant in our study could be that our study had a smaller sample size^11^.

We also recommend further research to investigate if the increase in plaque calcification volume in the intracranial part of internal carotid artery, reduces the risk of ischemic stroke. One could argue that a higher volume of calcification could stabilize the plaque consistent with previous literature^6–8^. But at the same time, if an increase in calcification volume also means an increase in total plaque volume, then the benefit of a more stable plaque in the smaller distal located vessels, might be omitted by an increased risk on ischemic stroke, caused by severe stenosis or higher total plaque volume.

The strength of our study is that we assessed the change in plaque calcification for both the extra- and intracranial part of the internal carotid artery in one session, which to our knowledge has not been done before and provides unique information for each location. Furthermore, we evaluated a symptomatic patient population, known with a TIA or minor stroke, and therefore prone to a recurrent stroke. Our results are relevant for this population since this can eventually lead to more patient-specific stroke management, potentially including information about the development of their atherosclerotic plaques during follow-up.

In this study, we also had some limitations. A threshold of 600 HU was used to separate calcification from contrast material in the lumen, instead of the 130 HU applied in non-contrast enhanced CT scans^19^. Therefore, by missing low-density MDCTA calcification (< 600 HU), there might be an underestimation of the calcification volume. However, since we used the same threshold at baseline and follow-up, we expect that the influence on our results is limited, although we could still have missed new low-density calcifications.

Another limitation was potential misclassification between stabilization and increase or decrease groups, caused by for example the use of different CTA scanners, artefacts (like blooming effect) or measurement errors. Blooming effect can lead to an overestimation of the calcification volume. This effect increases by an increased calcification volume. To deal with these potential misclassifications, we used a cut off value of 10% in relative volume change. Despite this correction and the reproducibility, some misclassification could still occur, since the cut off value of 10% is dependent on the baseline calcification volume. Patients with a low calcification volume at baseline could be easier misclassified, since the criteria of 10% change in calcification volume was met easier. Still, we expect not an evident selection bias, since we found lower calcification volume at baseline as the most important determinant of calcification progression.

The clinical relevance of our study is that it gives insight into the natural course of carotid plaque calcification. Our study also shows the possibility of calcification volume regression. More research has to be done to unravel this finding and find potential pathophysiological mechanisms that could explain regression in calcifications.

## Conclusion

We evaluated the natural course of atherosclerotic plaque calcification in the intra- and extracranial part of the carotid artery, over 2 year follow-up. Overall, a progression was seen in a narrow majority, which was associated with a lower calcification volume at baseline. Remarkably, we also found regression in calcification volume, which was associated with a higher calcification volume at baseline. Further research could investigate whether this change in plaque composition leads to a change in risk of (recurrent) ischemic stroke.

## Supplementary Information

Supplementary Tables.

## Data Availability

The data underlying this article cannot be shared publicly due to the privacy of individuals that participated in this study. Data will be shared on reasonable request to D. Bos, MD, PhD (d.bos@erasmusmc.nl).
