# Supplementary material for: Changes in intra- and extracranial carotid plaque calcification: a 2-year follow-up study
Source: Sci Rep. 2023 May 24;13:8384. doi: 10.1038/s41598-023-34223-7 (PMC10209086; doi:10.1038/s41598-023-34223-7)
Supplement: Supplementary file 1 — Supplementary Tables. [file 41598_2023_34223_MOESM1_ESM.pdf]

Supplemental Results

Supplemental Table 1. Differences between calcifications at baseline and two year follow-up (per artery)

	**ECAC n=156**	**ICAC n=140**
**Change in calcification volume (n (%))**		
Increase	72 (46.2%)	63 (45.0%)
Stable	31 (19.8%)	42 (30.0%)
Decrease	53 (34.0%)	35 (25.0%)
**Absolute differences (mm^3)**		
All arteries	1.0 [-29.3;30.8]	0.7 [-2.3;12.9]
Increase	23.0 [3.9;40.1]	12.9 [7.2;27.5]
Decrease	-36.4 [-78.4;-10.0]	-32.8 [-88.7;-11.5]

Increase and decrease of calcification volumes are defined as an increase or decrease of ≥ 10% compared to the baseline calcification volume. Patients with stabilizing calcification volumes either had calcification volumes of 0 at baseline and follow-up, or had < 10% increase of decrease of calcification volume compared to baseline.

Supplemental Table 2. Change in atherosclerotic plaque calcification for both carotid arteries per patient.

	**ECAC n=78**	**ICAC n=70**
Increase	34 (43.6%)	31 (44.3%)
Stable	6 (7.7%)	10 (14.3%)
Decrease	15 (19.2%)	12 (17.1%)

Values represent n(%).
